# Capturing emergency dispatch address points as geocoding candidates to quantify delimited confidence in residential geolocation

**DOI:** 10.1186/s12942-023-00347-2

**Published:** 2023-09-26

**Authors:** Christian A. Klaus, Kevin A. Henry, Dora Il’yasova

**Affiliations:** 1 North Carolina Central Cancer Registry, Raleigh, NC USA; 2 Department of Geography, Environment and Urban Studies, Temple University, Philadelphia, PA USA; 3 Division of Cancer Prevention and Control, Fox Chase Cancer Center, Philadelphia, PA USA; 4 Center for Social and Clinical Research, National Minority Quality Forum, Washington, DC USA; 5 Department of Community and Family Health, Duke University School of Medicine, Durham, NC USA

**Keywords:** Geocoding, Geocoding record linkage substitution, Health informatics, Residential geolocation, Attribute association, Residential address discriminant power, Residential geolocation discriminant power, Confidence in residential geolocation, Candidates of equivalent likelihood

## Abstract

**Background:**

In response to citizens’ concerns about elevated cancer incidence in their locales, US CDC proposed publishing cancer incidence at sub-county scales. At these scales, confidence in patients’ residential geolocation becomes a key constraint of geospatial analysis. To support monitoring cancer incidence in sub-county areas, we presented summary metrics to numerically delimit confidence in residential geolocation.

**Results:**

We defined a concept of Residential Address Discriminant Power (RADP) as theoretically perfect within all residential addresses and its practical application, i.e., using Emergency Dispatch (ED) Address Point Candidates of Equivalent Likelihood (CEL) to quantify Residential Geolocation Discriminant Power (RGDP) to approximate RADP. Leveraging different productivity of probabilistic, deterministic, and interactive geocoding record linkage, we simultaneously detected CEL for 5,807 cancer cases reported to North Carolina Central Cancer Registry (NC CCR)- in January 2022. Batch-match probabilistic and deterministic algorithms matched 86.0% cases to their unique ED address point candidates or a CEL, 4.4% to parcel site address, and 1.4% to street centerline. Interactively geocoded cases were 8.2%. To demonstrate differences in residential geolocation confidence between enumeration areas, we calculated sRGDP for cancer cases by county and assessed the existing uncertainty within the ED data, i.e., identified duplicate addresses (as CEL) for each ED address point in the 2014 version of the NC ED data and calculated ED_sRGDP by county. Both summary RGDP (sRGDP) (0.62–1.00) and ED_sRGDP (0.36–1.00) varied across counties and were lower in rural counties (p < 0.05); sRGDP correlated with ED_sRGDP (r = 0.42, p < 0.001). The discussion covered multiple conceptual and economic issues attendant to quantifying confidence in residential geolocation and presented a set of organizing principles for future work.

**Conclusions:**

Our methodology produces simple metrics – sRGDP – to capture confidence in residential geolocation via leveraging ED address points as CEL. Two facts demonstrate the usefulness of sRGDP as area-based summary metrics: sRGDP variability between counties and the overall lower quality of residential geolocation in rural vs. urban counties. Low sRGDP for the cancer cases within the area of interest helps manage expectations for the uncertainty in cancer incidence data. By supplementing cancer incidence data with sRGDP and ED_sRGDP, CCRs can demonstrate transparency in geocoding success, which may help win citizen trust.

## Highlights

- Confidence in patients’ residential geolocation becomes a key constraint of geospatial analysis for small areas.
- The presented novel methodology uses a concept of Residential Address Discriminant Power (RADP) as theoretically perfect within all residential addresses and its practical application, i.e., Emergency Dispatch (ED) Address Point Candidates of Equivalent Likelihood (CEL) to quantify Residential Geolocation Discriminant Power (RGDP) to approximate RADP.
- RGDP can be summarized for cases across a specific area (sRGDP) and serve as a threshold of confidence in residential geolocation.
- The analysis of data from the NC Cancer Registry showed wide variability of sRGDP between NC counties.
- The proposed methods for sRGDP apply to countries besides the US, specifically, those with a system of ED address points.

## Introduction

Citizens in North Carolina have voiced a wish for routine proactive cancer cluster scanning at sub-county scales to warn them of elevated incidence in smaller areas [1–3]. Similar concerns have been raised nationwide about assessing “timely, locally relevant information, including sub-county measures of health and associated factors” as such data help direct local public health actions [4–9]. In the states of Louisiana and New York, legislators required the health department to publish incidence data at sub-county scales [4]. In response, researchers at the Centers for Disease Control and Prevention (CDC) have proposed methods to routinely publish cancer incidence at sub-county scales for selected primary sites [4]. Their methodology proposes an assessment of cancer incidence in aggregations of enumeration areas (census tracts), [4, 5], with the sub-county regions selected based on rate stability and suppression thresholds [4, 5]. Their proposed methodology balances confidentiality and rate stability on one hand and granularity of geographic area on the other.

However, the proposed change in geographic scales for published cancer incidence represents a dramatic shift for the US state Central Cancer Registries (CCRs) and the public. Currently, proactive routine assessment of incidence for all primary tumor sites is conducted only at the county level. Under the proposed change, the incidence of selected primary tumors is to be proactively assessed for selected sub-county regions. With such a change, we foresee the following long-standing issues becoming acute. Identification of significantly elevated incidence rates in sub-county areas is likely to attract the attention of the citizens as more specific to their residence area [10]. At the same time, the smaller the geographical area, the more likely incidence rates are at the lower limit of rate stability (e.g., 16 cases in NC). At the threshold of rate stability, confidence in residential geolocation impacts confidence in incidence rates, because confidence in the patient's residential geolocation becomes a key constraint of geospatial analysis [11, 12] and is more likely to impact confidence in in incidence rates. As we reviewed the previously proposed methodologies, we found that most methods focus on quantifying accuracy in geocoding via relative or absolute positional accuracy metrics [11, 12]. In essence, the existing methodologies estimate the accuracy of the distance between an x,y-coordinate assigned during geocoding and the geographic feature that is used to approximate the position of a residential address. In contrast, we introduce a conceptually different method. We focus on quantifying address discriminant power of emergency dispatch (ED) address point(s), used to geocode patient address. These ED address points proxy an ***area*** (e.g., building footprint, parcel, postal code area) that delimits uncertainty in residential geolocation according to the evidence available at the time of geocoding. This enables us to quantify confidence in the four geographic units of analysis utilized for cancer epidemiology: enumeration area, parcel, building, and building unit, for each cancer case. ED address point domain presents the key source of residence proxy that is accessible to CCRs. ED address point data contain the domain of the vast majority of residential addresses and their corresponding geocode, attributes of which are not controlled by other statutory stewards.

Confidence in residential geolocation depends on the limits of data quality [5, 13] and stewardship. Quantifying confidence in patient address geolocation allows CCRs to demonstrate transparency to the public regarding the interpretation of the results [14, 15]. Given the interest in cancer incidence expressed by the public [13], we believe that sharing with the public the existing uncertainty in cancer record linkage, which is beyond the CCRs’ control, can strengthen the trust in the data reported by CCRs. It has been noted that, although citizen trust in local (state) government can be strongly determined by factors beyond the government's control [16], citizen trust relationships with government agencies are key for the agencies’ success in executing their missions [17]. There is no doubt that public trust in the integrity of research is extremely important [18].

Bearing responsibility for all uncertainties associated with multi-stewardship of the cancer surveillance data stream, CCRs are greatly motivated to quantify uncertainties that originate from non-CCR stewards. This can bolster trust relationships with citizens by ensuring transparency and accountability [15, 19]. To achieve this goal, we proposed a convention for attribute association-specific metadata in CCR record linkage operations [15]. Here, we propose repurposing ED address point data to quantify delimited confidence in residential geolocation, discussing the strengths and limitations of this approach.

## Methods

### Introduction to the proposed methodology

CCRs lack control over domain constraints of attribute sets that manifest the epidemiologic concepts of Person, Place, and Time (to a lesser extent) [15]. Limits to confidence in the data stem from the availability of information; some information is available to upstream stewards but is effectively missing to downstream stewards, which is typical of a disease surveillance data stream. Managed by upstream stewards, such attribute sets can be seen only as attribute associations (AAs) by downstream stewards like CCRs, meaning that downstream stewards have limited capacity to assess concordance or discordance between different fields of these attribute sets [14, 15]. Considering the cancer surveillance data stream, we proposed a convention of 15 AAs that enable and/or modify spatiotemporal relationships in cancer surveillance data as the basis for assessing delimited confidence in residential geolocation [14, 15]. We limited the number of AAs in cancer incidence data to just those for which an argument for quantifying confidence can be reasonably made. These AAs and their metadata store information about both the patient's residential geolocation and its uncertainty. ED address points present one of the most heavily used AAs for geocoding. Here, we focus on their address components, such as house number and street name, and their concordance or lack thereof.

The theoretical basis of our methodology is rooted in the discriminant power of residential addresses. The term *residence* implies the spatiotemporal finitude of the residential anthroposphere. This finitude is delimited with the domain of known residential addresses, which are designed to be perfectly discriminant, thus confer discriminant power. We introduce the term Residential Address Discriminant Power (RADP) as theoretically perfect within all residential addresses. ED data also have a certain discriminant power, which stems from the aspiration to the following objectives: (a) data including both object and attribute completeness as defined by Bleiholder and Naumann [20] and (b) having database integrity as defined by Motro [21, 22]. To best identify patients’ residential geolocation, CCRs employ geocoding record linkage against ED data. ED data are used to assign a geocode to the patient’s address by matching it to an ED address point. However, ED address points may not perfectly discriminate between residential addresses (e.g., missing sub-address). Therefore, residential geolocation (i.e., process of assigning residential geocodes) may incur a loss of discriminant power. We propose the term *Residential Geolocation Discriminant Power* (RGDP) to quantify losses of the discriminant power of a patient’s address during the process of geolocation.

### ED address point candidates of equivalent likelihood (CEL) to quantify RGDP for a single cancer case

Matching a patient address to a record linkage candidate most often yields the cardinality of the residential address to the ED address point candidate of 1:1, i.e., the best unique geolocation candidate for the address is identified. In such a case, there is no uncertainty in assigning a geocode to a residence. Uncertainty arises when input values (i.e., patient address) can be matched to more than one candidate in ED data (i.e., more than one address and/or residence).

A typical situation when ED address points do not perfectly discriminate between residential addresses is when ED address points are missing sub-addresses. This means, for example, that 12 ED address points are assigned to 12 sub-address-specific residences, but data does not specify the correspondence between address points and sub-address. As a result, these 12 ED address points have the same address but different geocodes; they become record linkage candidates to a patient address that matches one of the unspecified 12 sub-addresses. In this scenario, each of the candidates is equally likely to be a correct match; we denote the set of these match candidates as *candidates of equivalent likelihood* (CEL). Any of the CEL can be used to assign a geocode to this residence while incurring uncertainty or a loss of confidence in residential geolocation. We quantify the loss in RGDP as inverse to the number of CEL per linkage:

$$ {\text{RGDP }} = { 1}/{\text{CEL}}, $$

where RGDP is a fraction, ranging from > 0 to 1, and equals 1 when record linkage identifies a unique best candidate. RGDP decreases as the number of CEL increases, corresponding to the loss of confidence in residential geolocation. For some patient addresses, no ED candidates can be identified (for example, in residential areas undergoing construction to which ED address points have not been assigned). Then we deem CEL = 0. For such cases, the number of candidates still can be *estimated* as opposed to captured by enumerating existing ED CEL. This technique of estimation is the subject of future research topics, which is beyond the scope of this manuscript. Because the presented formula does not apply to cases with CEL = 0, for this manuscript, cases with no ED candidates are deemed to have RGDP = 0.

#### Using CEL to quantify confidence with geocoding record linkage substitution

When the best unique candidate cannot be identified, either one of the CEL or a less resolute proxy (e.g., postal code area centroid) can be chosen. These practices we term *geocoding record linkage substitution*. This term can serve as an umbrella for existing techniques in geocoding, used to avoid record linkage failure. Such techniques can include hierarchical geocoding and others that have been summarized and described in more detail by Goldberg [23] and by other authors [24–29]. Record linkage substitution presents a fundamental difference between the practices in geocoding and patient identity linkage. Record linkage produces a binary result for patient identity – success or failure. In contrast, geocoding produces results with degrees of success, depending on the size of the area of uncertainty and the number of CEL within the area. In some situations, a patient address has more than 1 set of CEL (aka ‘multi-match’), because more than one patient address component has uncertainty. Following the principle of information entropy maximization [30], these cancer cases must undergo geocoding record linkage substitution incurring less resolute geocodes until they can be geocoded with a single set of CEL. We illustrated different choices for record linkage substitution with a hypothetical example in Appendix 1 and illustrated how the principle of entropy maximization is a consideration in record linkage choices. In Appendix 2, we propose a convention for the record linkage substitution and calculating RGDP for cancer cases when ED address points candidates are derived from vector planimetric features, including street centerline.

### Summarized RGDP

To meet the requirements for protecting the confidentiality of patient data, CCRs cannot release RGDP for a single cancer case. These requirements can be better met when RGDP is summarized across cases, which we denote as sRGDP and propose the following calculation formula:

$$ {\text{sRGDP}} = \frac{{\sum \begin{gathered} N \hfill \\ i \hfill \\ \end{gathered} \frac{1}{CEL_i }}}{N} $$

In this formula, ${CEL}_{i}$ presents the number of ED address point candidates for the ${i}{\prime}s$ case; $N$ is the total number of cases in an area for a certain time period. As such, sRGDP for $N$ cases will diminish as the number of cases with multiple CEL increases. Cases with CEL = 0 contribute 0 to the numerator and 1 to the denominator. We propose to present sRGDP for all cases used to generate published incidence rates as a measure of the data quality for residential geolocation in a certain area.

### Proposed geocoding record linkage steps with computation of sRGDP Using a subset of NC CCR cancer cases

**Fig. 1:**
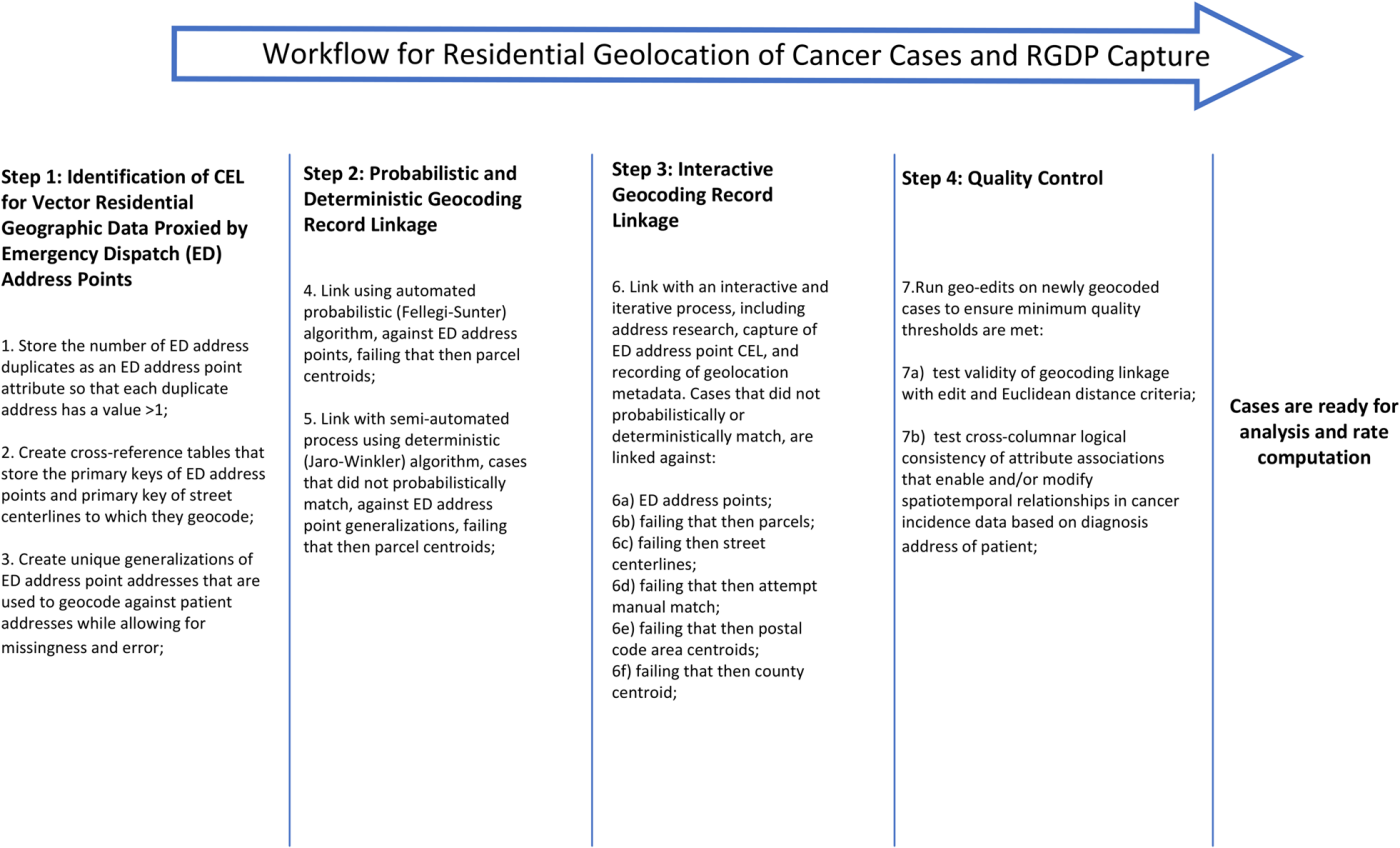
Workflow for Residential Geolocation of Cancer Cases and RGDP Capture

To demonstrate how our approach works with real data, we present the number of CEL per case using the recent data from the NC CCR that included all cancer cases reported to the NC CCR in the month of January 2022. We describe the workflow as consecutive steps in geocoding along with the quantification of uncertainty in residential geolocation. Below we describe these steps and summarize the workflow in Fig. 1.

#### Step 1: Identification of CEL for vector residential geographic data proxied by ED address points

We prepared for geocoding record linkage by identification of duplicates in the ED address domain (Fig. 1, Step1). Often, these duplicates present missing information about sub-addresses. We stored the number of duplicates as an additional meta-data field in ED address data. Further, we prepared for the use of the less resolute geographic reference data by creating cross-reference tables to link, for example, street centerline segments with the associated ED address points. Similarly, we created a cross-reference table to enumerate ED address points associated with each postal code area to prepare for using the postal code area centroid as a proxy.

Another part of the preparation included creating generalizations of all ED addresses using 2014 vintage NC ED data (Fig. 1, Step 1). At the time of our analysis the 2014 vintage ED address points [31] were the most recent statewide ED dataset. ED address generalizations are versions of an ED address from which one or more address components have been subtracted, following the principle of attribute relaxation described by Levine and Kim [32] and Goldberg [23]. For example, the postal code in the patient’s address does not match the combination of house number and street name. However, if the ED candidate address remains unique when we remove the postal code, we use that generalization for geocoding to make a match in spite of reported postal code being discordant. Approximately 80% of 2014 NC ED addresses were still unique without postal codes. We used unique address generalizations as a threshold for record linkage exhaustion because such a threshold is replicable and allows tolerance for error or missingness in patient address components during record linkage. Currently, there are no CCR community-specific conventions for record linkage exhaustion in geocoding of cancer incidence patient address, beyond verification of domain inclusion/exclusion during automated record linkage with commonly employed algorithms such as Felligi-Sunter [33]. Individual CCRs have the discretion to delimit the extent of their record linkage. On a monthly basis, 20–60% of NC CCR deterministic linkage matches involve ED address generalizations.

#### Step 2: Probabilistic and deterministic geocoding record linkage

Our procedure has been designed to mitigate additional expenses associated with the capture of CEL. By introducing quantification of confidence in residential geolocation, we are compelled to monitor record linkage productivity, which was not necessary for geocoding record linkage alone. In Table 1, we contrast different categories of geocoding productivity providing a framework to discuss this effort.

**Table 1:** Productivity of Geocoding Record Linkage and Quantification of Confidence in Residential Geolocation for Large Subsets of Cancer Cases

Procedure	Productivity		Exception handling
Probabilistic Linkage	100 K-1 M cases/hour*	High productivity per case	All cases that did not match are subject to deterministic linkage. No exception handling
Deterministic Linkage	100 cases/hour	Moderate productivity per case	Cases that did not match are subjects to interactive linkage. Some exception handling
Interactive Linkage	10 cases/hour	Low productivity per case	Cases that did not match are subject to geocoding record linkage substitution Greatest exception handling

*Depending on CPU resources

To maximize the number of cases geocoded at the higher productivity level, we used automated geocoding with a probabilistic algorithm to link a batch of patients’ addresses against ED address points and parcels, supplemented by the semi-automated match with a deterministic algorithm (Fig. 1**, **Step 2). In this research, we started with a probabilistic record linkage. Probabilistic record linkage utilizes an unsupervised classification algorithm designed to allocate field-specific weights, as demonstrated by Fellegi and Sunter [33]. These weights are determined by evaluating the level of concordance exhibited between the linked fields in order to compare the probability of match to probability of non-match. Because of its performance and scalability this approach has been extensively adopted in a variety of record linkage applications including geocoding. Generally, some addresses are not matched probabilistically and therefore, are linked with edit distance using a deterministic algorithm, which is a rule-based evaluation of string similarity.

To further minimize the number of cases requiring interactive geocoding, we deterministically linked patient addresses against unique ED address point address generalizations (Table 1). In this research, approximately 20% of deterministic matches involved address generalizations. The outcome of the probabilistic and deterministic linkage was determining the best ED address point candidate for a patient’s address or alternatively, capturing its CEL. The number of ED address point CEL for a patient address was derived from metadata (e.g., enumerated duplicate ED address points per address) or the number of ED address points corresponding to the street centerline, and stored in a cross-reference table. For parcel centroids, we determined CEL based on the geometric intersection between parcels and ED address points as this placement of address point relative to parcel is based on current standards and best practices [34]. Specifically, we considered CEL = 1 when a parcel geometrically contains one ED address point; generally, CEL was equal to the number of ED address points that coincide with a parcel.

Our strategy was to maximize the number of cases matched to the most address discriminant candidates which are either ED address point or parcel centroid. When all other accessible evidence was exhausted to identify a match to the ED address point or parcel centroid, we attempted to match the remaining addresses to a street centerline.

#### Step 3: Interactive geocoding record linkage

The remaining addresses did not match to an ED candidate automatically, because their match candidates did not meet probabilistic or deterministic thresholds of similarity. For these addresses, we conducted interactive geocoding attempting to further maximize matching them to an ED address point or parcel centroid. For example, the street name in the patient’s address could not be found in the street domain of the ED data. This happens when an apartment complex has street names within the complex, whereas ED address points are assigned only to the street that the complex faces. We attempted to use additional sources besides ED data (such as linking the patient’s name to the parcel’s owner’s name) to match an address to an ED candidate. Using additional sources of information allowed us to match addresses to either parcel centroid or street centerline interactively (Fig. 1, Step 3). When all other evidence for street-level or manual match has been exhausted, an attempt was made to geocode to the postal code area centroid. The addresses that contained a PO Box instead of a street address were geocoded to USPS branch offices.

We propose to recycle manually placed geocodes (code ‘08’ in the GIS Coordinate Quality metadata item [35]). The geocodes placed by CCR staff to proxy a residence (generally in areas distinguished by the absence of ED address points or parcel lot lines) can be recycled across cases. The meta-data associated with these geocodes communicates that their (x, y) coordinates are not included in the ED address point (x, y) domain, or other geographic reference data, at the time of geocoding. Manually placed geocodes generally indicate a building or parcel that has not had a planimetric feature assigned. As manual placement is a relatively expensive operation, its recycling increases productivity while quantifying confidence in residential geolocation.

The meta-data ideally includes post-geocoding referential integrity between the patient’s address and its CEL and/or storage of convex hulls that delineate the area of uncertainty in residential geolocation. The interactive process of encoding verification and establishing referential integrity with CEL is by far the most expensive part of geocoding with quantification of confidence. Consequently, it is important to verify that all possibilities of matching at a higher level of productivity have been ruled out.

#### Step 4: Quality control

We want to make a distinction between traditionally used quality control edits in cancer surveillance data and residential geo-edits. Traditionally, quality control of cancer surveillance data has been achieved through tests of cross-column logical consistency called edits [35]. This is done in part to maintain a minimum threshold of comparability of data quality across subsets of cases. Application of these principles to residential geolocation necessitates residential geo-edits, i.e., the test for logical consistency of the domains of data fields that enable and/or modify spatiotemporal relationships in cancer data (Fig. 1, Step 4). These are controlled by stewards other than CCRs [15]. Because of this, meeting a minimum threshold of comparability across subsets of cases requires disproportionately more edits as compared to the domains controlled by CCR data domains. In part because they are verifying geography, residential geo-edits tend to be more numerous than edits for CCR-constrained attribute sets, on an assessed field basis.

Examples of residential geo-edit are tests for consistency between nested census enumeration areas (i.e., US census block group, tract, and county). Geo-edits must verify, for example, that certainty of census tract [35] corresponds to levels of geocoding record linkage substitution as indicated by other meta-data. In addition to testing for consistency in cross-column domain and/or metadata fields, residential geo-edits also need to scan for discordance between reference data, e.g., whether two reference datasets disagree on the county associated with a given address point.

For this research, we used more than 500 residential geo-edits, many of which are specific to NC. These geo-edits included a core set that is ostensibly applicable to all US states to meet minimum thresholds of confidence in residential geolocation. Two of these are needed to screen for false positive matches in address and geocode. Cases exceeding a minimum threshold of edit distance between original and matched address, or Euclidean distance between geocode and centroid of original postal code area, were interactively reviewed, their values changed, or else were assigned override meta-data based on patient address research. Further, we scanned for parcels and building footprints, that span a census enumeration area boundary, when parcels and building footprints are used to derive ED geocode. Of these, a small proportion were address points with missing sub-address on either side the of enumeration area boundary, or the ED address points sharing a common geocode (e.g., high-rise buildings with multi-residences). In these circumstances, the relationship between address and enumeration area based on geometric coincidence breaks down. We quantified the loss of confidence in residential geolocation that this scenario incurs.

The disproportionately large number of geo-edits, relative to the fields they assess, necessitated cost management. The larger the number of edits, the more time was needed to ensure that they are mutually exclusive (non-redundant). Another important consideration was runtime performance. We have managed the costs of geo-edits by running them at off-peak times.

#### Sources of uncertainty in geolocation of residence at diagnosis

We broadly divided the origins of uncertainty in residential geolocation between two sources of addresses– from ED address point data and from demographic data (patient address). Patient address is authored the by patient and/or medical facility on behalf of the patient. This distinction is important to citizens impacted by the publication of cancer incidence rates at the sub-county scale because it ostensibly allows the concerned citizens to follow up with the organizations responsible for the uncertainty in the data used to produce the sub-county incidence rates.

We used the meta-data generated in Step 1 to identify cases for which uncertainty in residential geolocation stems from missing attributes in ED data. When CEL = 0 but a residence was apparent in orthophoto, then ED candidate points were effectively missing; the uncertainty in residential geolocation for such cases was attributed to the current vintage ED data. Missing address points may be an artifact of data vintage as their missingness is apparent during analysis but not necessarily at a later date. Note, assignment of uncertainty origin to ED stewardship for cases with CEL = 0 may be at best preliminary. If the uncertainty could not be traced to ED data, it is assumed to stem from the patient or medical facility.

### Statistical analysis

To illustrate how enumeration areas differ in confidence in residential geolocation, we calculated sRGDP for cancer cases by county. In January 2022, medical facilities reported cases that were geocoded to 74 out of 100 NC counties. In addition, we assessed ED data quality for these 74 counties as a general measure of ED data quality, using ED meta-data and the cross-link files that were created in preparation for geocoding. Specifically, we identified the number of CEL for each ED address in the 2014 version of the NC ED data and calculated ED_sRGDP for each county.

We explored whether each measure – sRGDP and ED_sRGDP – differs between rural vs. urban counties, using the Wilcoxon test. The categorization of counties as rural or urban was according to US Census urban/rural designations as of 2010. We hypothesized that confidence in residential geolocation of cancer cases correlated with the quality of ED data as assessed by ED_sRGDP. To test this hypothesis, we determined the degree of correlation between sRGDP and ED_sRGDP by county, using the Pearson correlation coefficient. We also hypothesized that sRGDP within a certain county is lower than ED_sRGDP in the same county due to added uncertainty, originating from patients and or medical facility reporting. To evaluate formally whether sRGDP and ED_sRGDP differ, we combined them in a regression model as a continuous outcome with two categorical predictors: cases-related vs. ED-related and rural vs. urban. The negative value of the beta-coefficient for case vs. ED data and the p-value for the coefficient < 0.05 was interpreted as evidence in support of the hypothesis.

## Results

The results of the proposed geocoding are presented according to geocoding productivity (Table 2). We identified a total of 5,807 cancer cases reported to NC CCR during the month of January 2022. Using probabilistic and deterministic algorithms, we identified CEL for 5,424 and no CEL for 86 cases. Based on our previous experience, we expected that this step of record linkage matches > 70% of patient addresses to either a single best candidate (CEL = 1) or to an address with multiple CEL (CEL > 1). In agreement with our expectations, 86.0% of cancer cases (n = 4,994) were batch-matched either to the best unique ED address point (84.9%) or to an address point that has multiple CEL as determined from meta-data. The number of CEL for the 78 cases is derived from enumerated duplicates: a total of 1,023 CEL for 78 cases were identified, with an average of 13 CEL per case. The loss of confidence in residential geolocation for the 78 cases with multiple CEL is attributed entirely to the ED data.

**Table 2:** Results of Geocoding with Uncertainty Capture by CEL: NC CCR data for all cases of cancer reported January 1–31, 2022

Geographic Reference Data	Cases					Sources of Uncertainty in the Geolocation of Residence at Diagnosis		
	Total N = 5807 (%)^a^	Linkage results						
		Best unique E911 address point Candidate CEL = 1	No E911 address point candidates CEL = 0	Multiple E911 address point candidates CEL > 1		E911 Data		Patient/Medical facility
		N (%)^a^	N (%)^a^	N (%)^a^	Total CEL^b^ (CEL per case)	CEL > 0	CEL = 0	
Geocoding using probabilistic and deterministic algorithms								
ED Address Point	4994 (86.0)	4916 (84.9)	N/A	78 (1.3)	1,023 (13 CEL/case)	100%	0	0
Parcel Centroid	253 (4.4)	180 (1)	70 (3.0)	3 (0.0)	6 (2 CEL/case)	4%	96%	0
Digital Street Centerline	80 (1.4)	3 (0.04)	16 (0.2)	61 (0.76)	4714 (77 CEL/case)	35%	20%	45%
Interactive geocoding								
ED address point	389 (6.7)	378 (6.5)	N/A	11 (0.2)	101 (9 CEL/case)	100%	0	0
Parcel centroid	23 (0.4)	4 (< 0.1)	19 (0.3)	0 (0.0)	N/A	17%	83%	0
Digital street centerline	19 (0.1)	1 (< 0.1)	6	12 (< 0.1)	492 (41 CEL/case)	24%	32%	44%
Manually placed	2 (0.2)	0	2	0 (0)	N/A	0%	100%	0
Postal code area centroid	31 (0.3)	0	0	31 (0.3)	456,847 (24,045 CEL/case)	8%^e^	0%	92%
Post office box location^c^	5 (0.1)	0	N/A	5 (0.1)	24,699,660 (4,939,932 CEL/case)	6%^e^	0%	94%
County centroid^d^	9	0	0	9	781,683 (86,851/case)	1.5%^e^	0%	98.5%
State centroid^d^	0	0	0	0	0	N/A	N/A	N/A
Not Geocoded	2 (< 0.1)	N/A	N/A	N/A	N/A	N/A	N/A	N/A

^a^Percent of total N = 5807

^b^Based on 2014 E911 data

^c^Since the E911 CEL of a Post Office Box geocode can span an entire state at a minimum, we have used the E911 address point count for the state of NC

^d^For the county centroid and state centroid, a total number of E911 address points within a county or state are used to derive the number of CEL

^e^The portion of E911 address points within the area of uncertainty which is not discriminant due to missing sub-addresses

We continued the probabilistic batch-match for the remaining addresses by matching them to the best parcel site address and geocoding using parcel centroid. In our sample, 4.4% of all cases (n = 253) could be matched to a parcel centroid (Table 2). The majority of these cases (n = 180) were matched to a unique best candidate, and 3 cases were matched to parcel centroid with multiple CEL (2 CEL per case on average). Some parcels did not coincide with any existing ED address points: a total of 1.2% of all cases (n = 70) in our data (Table 2). We consider ED address point CEL = 0 for these cases. The identified uncertainty for 73 cases is due to ED data, with the uncertainty for 96% of these cases attributed to a lack of ED address points associated with the patient address (CEL = 0).

There were 80 cases (1.4%) that were matched to the street centerline. The majority of these patient addresses (n = 61) had multiple CEL, with an average of 77 CEL per case. This situation arises when, for example, the house number is missing from the patient's address. Three cases were matched to a centerline segment that was associated with one ED address point. Sometimes, the streets are small with only one ED address point, presumably corresponding to one residence. There were 16 cases matched to street centerline that was not associated with ED address point CEL (CEL = 0). Partitioning the origins of uncertainty for the 77 cases, we found that 35% of uncertainty is due to duplicates in ED data, 20% is due to a lack of ED address points, and 45% of uncertainty is due to missing information in the reported patient address.

For the remaining 480 patient addresses, we attempted to geocode interactively (Table 2). The majority of these addresses (n = 389) were matched to a single best ED candidate (n = 378) or an address with multiple CEL (n = 11). Also, 23 and 19 addresses were matched to parcel centroid and street centerline, respectively. For 2 addresses, we manually placed the geocode. These addresses were in residentially emergent areas, where parcels lacked street addresses or streets lacked street names. After record linkage exhaustion, 31 addresses were geocoded to postal area centroids, 5 to postal office box locations, and 9 to county centroids. In this subset of cases, none had to be matched to a state centroid, an option when only the state of residence can be identified. Finally, 2 addresses could not be geocoded because either their state or country of residence could not be identified.

As the resolution of geographic features (to which an address is geocoded) decreased, the number of CEL and the uncertainty in residential geolocation increases (Table 2). The lowest number of CEL was found for parcel centroids, followed by the number of CEL for ED address points. The number of CEL for both of these record linkage substitution choices was below 20. A medium number of CEL were identified for street centerline (CEL < 100). Other record linkage substitutions – postal area centroid, PO Box location, and county centroid – had the number of CEL in the order of 10^5^–10^6^. Accordingly, a dramatic increase in the uncertainty of residential geolocation occurred with the increases in the number of CEL.

Summarizing the origins of uncertainty in residential geolocation, we note that for the cases matched to the ED address point and cases with CEL = 0, all the uncertainty stems from ED address point data. For the cases matched to parcel centroid, a large portion of uncertainty also stems from ED data. The contribution of patient/medical facilities to the uncertainty increased when cases are matched to street centerline and became the major source when cases were matched to area centroids.

The range variability by county was 1.6-fold (0.62–1.00) for sRGDP and 2.8-fold (0.36–1.00) for all ED_sRGDP. The summary measures of confidence in residential geolocation were lower in rural counties for both cancer cases and ED address points (p < 0.05). In fact, sRGDP correlated with ED_sRGDP, with the Pearson correlation coefficient of 0.42 (p = 0.0002). Using a regression model, we evaluated whether sRGDP is lower than ED_sRGDP after adjustment for the rural–urban difference and found that this difference was not significant (p = 0.24). At the same time, on average both sRGDP and ED_RGDP were lower in rural vs. urban areas by 0.044 (p = 0.02).

Reporting these results, we note several exceptions to using ED address point as a proxy for a unit or a building:

- ED address points can share a single geocode (stacked) as found, for example, in high-rise buildings. This scenario accounts for 2.3% of our sample of cancer cases where all the stacked E011 points constitute CEL, determined by the automated query.
- ED address points can lack 1:1 cardinality with apparent residence on orthophoto. This scenario accounts for 0.1% of cases in our sample, with candidates interactively determined.

There were also exceptions for calculations of sRGDP when there was a disagreement among upstream statutory stewards of address (ED data stewards, US Postal Service, US Census Bureau) regarding correspondence between address and county (1.0% in our sample of cancer cases). These disagreements were resolved in favor of the address-county association specified by ED data.

## Applicability of the proposed methodology to cancer registries outside of the USA

The proposed methodology is based on ED address points and therefore, is highly applicable to Cancer Registries in countries with emergency dispatch capacity. Emergency dispatch is a widespread and growing practice worldwide [36]. Recent programs with successful accreditation certifications include Trinidad, New Zealand, Malaysia, Lithuania, Qatar, Ireland, Wales, Austria, China, Brazil, Switzerland, and Canada among others [36]. The best-financed programs, generally, have ED address points, including Australia [37, 38], UK [39, 40], Canada [41, 42], and France [43], with citizens in other countries aspiring to develop these data [44]. In countries with emergency dispatch capacity, national postal agencies generally play a role in stewarding selected components of residential addresses, with local addressing authorities generally stewarding the remainder [37, 39, 45, 46]. Inevitably multiple stewardship of address components with different update cycles produces uncertainty in residential addresses. Our methodology may apply to these countries, where it is possible to use ED address points as CEL to quantify confidence in residential geolocation. Similar to the USA, funding of emergency dispatch services across provinces is uneven, for example, in Canada [42]. Using our methodology, Cancer Registries can contrast the extent of uncertainty in the residential geolocation of cancer cases between different areas.

## Discussion

We have presented a series of steps and metrics to utilize ED address points for quantifying confidence in residential geolocation of incident cancer cases. Prior research recommends that geocodes are assigned only on the condition that the associated uncertainty is quantified [47–49]. A longstanding hope has been that CCRs, researchers, and the public can use quantified metrics – arguably more objective and specific than narrative descriptions of data limitations – to understand the fitness of residential geolocation data [50]. In fact, quantification of uncertainty is key to producing the feeling of confidence [51], which is necessary for trust. Using AAs to manifest the epidemiologic concept of Place in CCR data implies that quantified confidence in residential geocoding record linkage is just as important as residential geocoding outcomes by providing verification and helping to earn trust in cancer incidence data. Citizens may discover that their sense of well-being and/or property values are more affected by the publication of cancer incidence at sub-county scales as compared to the current practice of publishing county incidence rates. Operationally, quantified confidence enables comparability of data quality for subsets of cases, which is important to assess when elevated incidence rates are detected.

ED address points are the best choice in our proposed AA convention [15] to summarize uncertainty in residential geolocation effectively. Theoretically, the domain of residential addresses presents closed-world data (all real-world entities represented in data) and is perfectly discriminant by design, such that residential address discriminant power is always equal to 1. At any given time, the ED address point address domain generally approximates the domain of existing residential addresses. ED candidates are preferential for assessing the uncertainty in residential geolocation as opposed to parcel site address due to two primary differences between them. First, parcel site addresses are not generally required to be unique, because the primary identification of parcels is not a site address but its unique identifier (primary key). Second, ED addresses are subject to continuous data quality control as address errors are continuously corrected based on feedback from emergency dispatchers. In contrast, such a quality control loop does not commonly exist in the parcel site address data. At the same time, parcel centroid geocodes are often incorporated into ED data as a cost-saving measure. When this happens, parcel site addresses become subject to ED quality control. As a result, much of the parcel address domain is eventually incorporated into the ED address domain. However, when ED address points are derived from parcel centroids, they may only approximate the geolocation of residence when the correspondence between the ED address point and residence is 1: > 1. Because of the existing aspiration of some ED stewards to attain 1:1 correspondence between E11 address points and residences, this uncertainty is likely to be reduced in the future. We believe that such a trend increases the utility of ED address point CEL for summarizing uncertainty in residential geolocation.

**Fig. 2:**
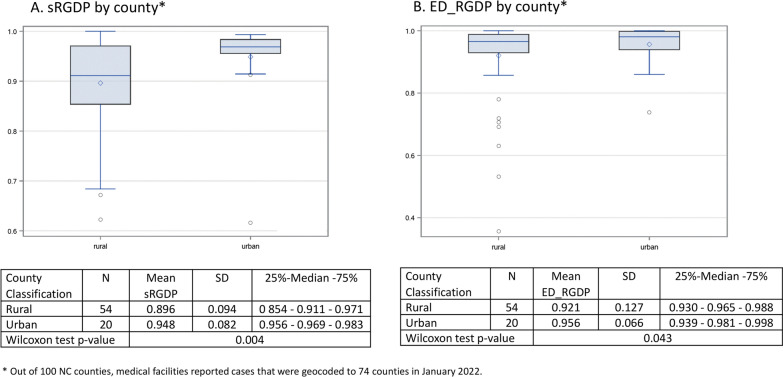
Comparison of sRGDP and ED_RGDP between rural and urban areas

We demonstrated that the fitness of ED data (ED_RGDP) and confidence in residential geolocation of incidence cases (sRGDP) vary widely between the areas (e.g., counties used in our analysis) (Fig. 2). For example, we found one county with an ED_RGDP of 0.36, whereas 75% of the counties assessed had ED_RGDP ≥ 0.93. A low ED_RGDP suggests problems with ED data. Such problems can include underinvestment in geographic reference data in general as well as erroneous extraction-translation of address data. Similarly, in one county, sRGDP was 0.62, whereas 75% of counties had cases of sRGDP ≥ 0.88. These summary metrics can be used to establish a threshold for confidence in residential geolocation, helping to exclude either a set of cases or specific areas from analysis due to poor data fitness. We believe that such quantification is also necessary to support the interpretation of uncertainty by citizens impacted by the publication of increased incidence rates at sub-county scales. For example, when sRGDP is 0.6 and ED_RGDP is 0.4, it demonstrates to citizens a poor quality of residential geolocation data effectively and concisely. It would be ideal to use sRGDP as a benchmark for the comparability of confidence in residential geolocation between different sets of cancer cases.

As noted earlier, citizens have indicated a wish for proactive cancer incidence scanning at sub-county scales as a means of warning them [1, 2]. In that, citizens do not necessarily wish to wait for a consensus on understanding the disease or its causal factors [52, 53]. What constitutes a warning then depends in part on confidence in residential geolocation, which as we demonstrated can vary significantly (Fig. 2). Thus, quantifying confidence in residential geolocation presents an important tool for CCRs to earn citizen trust by demonstrating when a low data fitness affects confidence in the incidence rates, i.e., the uncertainty that CCRs cannot control. This is especially important since CCRs anticipate more challenges to their ostensible role as the primary arbiter of uncertainty in cancer incidence data due to the increased number of inquiries in the wake of the routine publication of cancer incidence rates at sub-county scales. Besides the sRGDP estimates, CCRs can also demonstrate the origins of uncertainty in residential geolocation, providing actionable information for citizens to follow up with organizations responsible for this uncertainty. While CCRs are statutorily obligated to collect, curate, and disseminate aggregated cancer incidence statistics, their downstream position guarantees at least some uncertainty in residential geolocation in their casefiles that they cannot resolve with record linkage. From a public relations perspective, CCRs have a strong incentive to quantify uncertainty in published rates to demonstrate the quality of the data. In Table 2, we showed that most uncertainty in residential geolocation of cancer cases is due to ED data fitness when patients’ addresses are matched to an ED address point or a parcel centroid. However, when a patient address is matched to a street centerline, the loss of confidence due to missing or erroneous address data originated mainly from patient or medical facility records (~ 45% in our data as shown in Table 2). Patient/medical facility stewardship of the address data is also the predominant source of uncertainty when a patient’s address is geocoded to an area centroid other than a parcel (Table 2).

Our discussion of RGDP would not be complete without pointing out differences between using RGDP as a measure of discriminant power of address or geocoding record linkage and Shannon entropy [54, 55], which is commonly used in record linkage based on patient’s identity. Shannon entropy measures the discriminant power of a patient’s identity attributes to determine the lowest number of variables necessary for a successful match of patient records between several databases [54, 55]. As noted earlier, the outcome of this type of record linkage is binary – success or failure. Shannon entropy is calculated even before a record linkage occurs to maximize success. In geocoding the patient’s address, the outcome has a degree of success with RGDP reflecting the uncertainty of geocoding. Thus, Shannon entropy and RGDP are measuring different types of discriminant power. We note another principal difference between Shannon entropy and RGDP. Whereas the domain of residential addresses presents closed-world data (all US residential addresses are known) and is perfectly discriminant by design, the patient’s identity domain is not necessarily designed to be discriminant, with the most common example being high-frequency names. No database includes all individuals residing in the US at any given time, therefore, the domain of patients’ identities constitutes open-world data. Consequently, the measurement of the discriminant power of the name “John Smith” using candidates from one or another database does not make sense. A calculation of Shannon entropy for the variable “social security” vs. the combination of variables “first name”, “family name”, “sex”, and “age” can provide important information on whether or not extra effort should be undertaken to obtain social security number in addition to the information existing in the database. Calculating RGDP for an ED address point using CEL makes sense because ED address points present the reference data approximating all residential addresses that are designed to be unique.

In the past few years, the standard for geocoding quality has been transitioning, as some SEER-funded CCRs have pursued geocoding to enable the publication of incidence rates at the census tract level [56, 57]. In this context, the quality of geocoding of incidence data is considered as the percentage of cases with street-level matches. NCI SEER-funded CCRs are granted extra funding to search for missing patient demographic data including street address, which helps to increase the number of cases with street-level geocoding to 97.5–98% [56, 57]. The 98% level may represent a ceiling, with the remaining 2% including homeless patients and others whose addresses are not easily found. In 98% of cases, we expect at least a portion of the street-level matched addresses to have more than 1 CEL. In our example of cases reported in January 2022, 97% of new cases were matched to street level, but only 92% have RGDP = 1. Thus, the percentage of street-level matched addresses does not provide the full picture of the uncertainty in residential geolocation. Not having additional funding for case follow-back, NC CCR nevertheless has been able to achieve street-level match rates between 93–96% for new cases every month. By providing sRGDP, we better demonstrate the uncertainty in residential geolocation as compared to reporting the percent of the street-level match alone.

It is typical for CCRs to receive requests for frequent updates on cancer incidence in areas where citizens have demonstrated concern. During periods when citizens experience collective anxiety related to elevated incidence rates, CCRs can effectively address the situation by providing summary verification of confidence in residential geolocation as potentially the most important determinant of uncertainty in cancer incidence data. Such verification will inevitably require rapidly executable code that generates AA-specific, XML (extensible mark-up language)-encoded metadata because XML enables specification of AA. This is needed for any data which is at least partially authored by the CCR as well as for the data not authored by the CCR when stewards upstream of CCRs do not provide metadata. If upstream stewards provide metadata, CCRs need the capacity to harvest and incorporate the upstream metadata into their own and will have to utilize commonly accepted standards, such as those published by US Federal Geographic Data Committee [58]. Unsettled is the issue of AA-specific conventions for record linkage exhaustion. Conventions for record linkage exhaustion are necessary because the extent of referential integrity (i.e., whether just primary keys of geographic reference data, or whether in addition ED CEL are captured) depends in part on the extent of exhaustion. At a minimum, sRGDP and ED_RGDP are key indicators of confidence in residential geolocation suitable for a dashboard that may be easily interpretable by citizens, helping earn their trust. Further, the metadata will need to summarize the extent of cases with CEL = 0, which we demonstrated as a percentage of cases in Table 2. These cases will have to be revisited in the event of sub-county investigation with the expected newer vintage of ED data. Although there is no convention for record linkage exhaustion in address research, some methods present examples of non-scalability across a large group of cases. Some cases can be linked, for example, to scanned property deeds (i.e., “deed research”) to link patients and thus tumors to addresses and/or geocodes, a practice that is not scalable to the entire case file. Because these are time-consuming methodologies, summarizing in the metadata the percentage of such cases will display to concerned citizens the details of the extent of record linkage exhaustion for a subset of cases.

For incidence rates at sub-county scales, the relative importance of a single case can increase substantially compared to rates at county scales. As geographic reference data are updated, the geolocation of some cases can change between sub-county regions. Therefore, assessment of referential integrity between consolidated tumor records and geographic reference data (including ED CEL), becomes a key capacity for CCRs. Such post-geocoding ongoing verification is a continuous task, needed for all geographic reference data, specifically for residentially emergent areas and even for ED address points that are updated in residentially static areas. In other words, a capacity for rapid, recurrent and replicable assessment of referential integrity inevitably becomes key to maintaining confidence in residential geolocation across the cancer case file, and not just because new cases are perpetually being added. This is especially true during periods of collective citizen anxiety when the public requests frequent updates on reports of incidence rates or case counts in small areas.

Both the metadata and the metrics incur additional responsibility and expense to CCRs. It is primarily to mitigate the added expense that we deterministically link against ED address generalizations, the effect of which is to convert relatively expensive interactive linkage to less expensive deterministic linkage for many cancer cases. This linkage is undertaken after validation of postal address but prior to patient address geocoding linkage exhaustion, so that some but not all patient address aliases and/or address components are corrected, and thus will not necessarily guarantee the verification of ED address domain exclusion for all relevant cases. However, it is still effective in that regard, giving the CCR assurance that investing time in interactive geocoding of remaining cases based on domain exclusion is well spent. The proposed procedure reduces the number of candidates that must be interactively reviewed had the ED address generalizations not been leveraged. We also take this approach because it allows us to screen out-of-state street addresses mistakenly paired with the in-state city and postal code areas. Well over 99% of patient street addresses are clearly included in or excluded from the ED address domain, with only a very few remaining ambiguous at record linkage exhaustion.

The additional expense can also be mitigated because the tradeoff between geocoding completeness and confidence in residential geolocation is made evident by sRGDP. This argument is supported by the fact that the number of databases used for address research and/or geocoding is limited. We showed that there are 15 core reference databases, commonly used by CCRs for residential geolocation [15]. However, there is no formal consensus on what constitutes record linkage exhaustion for these databases. Based on published best practices [14, 23], we expect that the CCR community will be amenable to forging a consensus on the extent of the additional scope of work and expense (which is directly proportional to record linkage exhaustion) to quantify confidence in residential geolocation. Theoretically, there is an upper limit of the time needed to establish referential integrity (or reasonable approximation thereof) between all 15 reference databases and a patent address, which is likely to be more than CCRs can afford to undertake. However, we demonstrated that at least delimited confidence in residential geolocation can be produced using a subset of reference data, i.e., ED address points, parcels, and street centerlines.

The cost associated with quantified confidence in record linkage is further mitigated by complimentary savings provided by its flexibility in data management. As noted earlier, with the upcoming publishing of sub-county incidence rates, the requirements for assessing uncertainty in the residential geolocation of each case increase. For example, if the estimated chance of a false positive match is greater than 50% and evidence is entirely captured with CEL, then the match can be made because the extent of uncertainty is delimited, whereas ordinal or nominal data would not support the decision to the same degree. With the uncertainty quantified by CEL, the analyst has a tool to communicate that all the uncertainty is captured (helping to win the trust). Also, if the geographical reference data remain unchanged, likely, these cases do not have to be audited in the future interactively, saving the effort. With referential integrity between CEL and a patient address, the auditing can be automated due to the existing metadata. In contrast, if nominal or metadata are used to document uncertainty in residential geolocation (even when geographic reference data remain unchanged), the likelihood of interactive auditing with low productivity increases. Furthermore, geocodes with uncertainty can under certain circumstances be effectively ‘recycled’, because, for example, the addresses with geolocation uncertainty in high-density areas tend to be repeated among patients (e.g., patients from the same apartment complex without sub-addressed ED address points). Thus, CEL metadata for ED address points helps to save effort on interactive geocoding.

We have noted throughout the manuscript the public interest in cancer incidence data [1, 2]. The surveys clearly demonstrate the interest, indicating that citizens *want* to believe in science [59] where cancer is concerned, and thus are willing to accept the consensus of the scientific community and state agencies. This conclusion is supported by the US congressional votes consistently indicating bipartisan support for the scientific method to advance understanding of the disease [60]. Given this, a reasonable goal is to enable citizens to become co-arbiters of uncertainty in CCR incidence data, helping to adjust their expectations. Summarizing this research, we propose the following concepts as a first step towards accomplishing this goal. The proposed concepts in Table 3 stem from cancer case abstraction standards and best practices in cancer epidemiology, information science, and the spatial analysis of cadastral data, as well as from a range of concepts specific to our proposal (in no particular order). Although summarized as distinct, the proposed concepts are intimately connected and cannot be separated in the application.

**Table 3:** Concepts for quantifying uncertainty in residential geolocation of cancer incidence data

Concept	Example
Leveraging discriminant power of residential address to quantify confidence in residential geolocation	We propose to use of CEL and sRGDP
Enabling citizen understanding of quantified confidence in residential geolocation in cancer incidence data	We propose that uncertainty metrics be no more complex than necessary. At least some evidence (e.g., ED address points) should be available to citizens for independent verification
Using the principle of maximizing information entropy during geocoding record linkage to ensure comparability of confidence in residential geolocation across the subset of cases	Geocoding against ED address generalizations helps to ensure that a geographic area of uncertainty corresponds to the extent of accuracy and precision of address components
Differentiating between the domains of emergency dispatch address point and residential address	We clarify that the ED address domain is not necessarily as extensive or discriminant as the residential address domain at any given time
Clarifying the role of attribute associations and data stream stewardship in earning citizen trust	We quantified uncertainty due to ED reference data vs. patient/medical facility errors
Adding metadata to geographic reference data to facilitate quantification of confidence in residential geolocation	We add value to ED address point data by storing the CEL count of each duplicate address, so that it is easily accessible. This reduces the number of cases for which CEL has to be interactively identified
Delimit responsibility for quantification of confidence in residential geolocation by CCR as a downstream steward	A proposed convention delimiting AA that enables and/or modifies spatiotemporal relationships in CCR data [15]

## Limitations

There are limitations to the proposed methodology. Any constraint on the accuracy of the case count affects the accuracy of sRGDP. Currently, there is no consensus within the CCR community on a set of methods or negotiation protocols to determine the US state at diagnosis when evidence is unclear or missing. Such consensus is necessary for de-duplicating cancer cases across (US) states, and in particular, this impacts cases missing demographic data, especially (US) state or country of origin. For example, in recreationally attractive areas a large percentage of residences are vacation homes. In these areas, there are at least some cancer cases with unclear US state of residence and missing address of diagnosis, as reported by a medical facility. CCRs are given instructions to be conservative regarding the exclusion of cases whose state of diagnosis is unclear so that in the assignment of state of diagnosis to a tumor, the risk of false positive assignment is perceived as less than the risk of false negative assignment. As a result, the summary number of cases in all CCR files may be overstated relative to that in a hypothetical case file that is de-duplicated across the US states. At the limits of rate stability, this distinction becomes important, as such cases may not belong to a single state, thus impacting the validity of confidence in incidence rates at sub-county scales in particular. The proposed metrics of confidence in residential geolocation cannot address this situation. This can be addressed only through a consensus of methods to comprehensively de-duplicate cases across the states.

Another limitation of the proposed methodology is the lack of dedicated funding. Quantifying confidence in residential geolocation is currently outside the scope of funded activities of both CDC and NCI-funded central cancer registries. We, therefore, acknowledge a need to develop conventions and best practices in record linkage while quantifying confidence in data. In the future, we expect the development of self-learning artificial intelligence-based software to scale our approach. Additional funding can be justified by creating the key capacity for rapid assessment of referential integrity between consolidated tumor records and various geographic reference data in situations of collective citizen anxiety. If this capacity were considered critical to meeting requirements for collective citizen trust, then the consideration of its attendant expense becomes a different discussion.

## Conclusions

Cancer incidence data rely on patients’ residential geolocation at the time of diagnosis in determining the number of cases arising within a certain geographical area during a defined time period. As the publication of sub-county scale incidence rates continues to expand, uncertainty in CCR data stemming from residential geolocation of patient addresses becomes more difficult to assume away. We present a methodology to produce simple metrics – sRGDP – to capture the uncertainty in residential geolocation for a subset of cases via leveraging ED address points as CEL. Two important facts demonstrate the usefulness of sRGDP as area-based summary metrics: (1) sRGDP for cases and the quality of ED address point addresses expressed as ED_sRGDP varied widely by county; (2) both measures were lower in rural counties than their urban counterparts. Thus, low sRGDP and ED_RGDP within an area of interest help manage expectations for the uncertainty in cancer incidence data. We also demonstrate the uncertainty in address geolocation within the ED address point domain, emphasizing the role of the upstream stewards, thus, delimiting the responsibility of CCRs. The importance of upstream stewardship follows from the observed correlations between sRGDP and ED_RGDP. Overall, there were more cases (n = 235, 4.0%) with uncertainty stemming from ED data than cases with uncertainty stemming from incorrect patients address (n = 88, 1.5%). Also, sRGDP is more informative compared to the existing standard of geocoding quality for tract-level publication of incidence rates (i.e., percentage of geocodes at street level). We demonstrated that in our sample of cases, statewide 97% were street-level geocoded but only 92% had the best unique candidate for street-level geocode, with 5% having more than 1 CEL. By supplementing cancer incidence data with sRGDP and ED_RGDP, CCRs can showcase how geocoding becomes more comprehensive and rigorous with the addition of quantified confidence in residential geolocation. This diligent approach could potentially enhance citizen trust. Quantified confidence – and the referential integrity to CEL – can save time in the future, by enabling automated verification of patterns of coincidence between CEL and, for example, newly published enumeration area boundaries. Future research will focus on AAs other than ED address points and on the differences between the methods of capture vs. estimation of uncertainty associated with specific AA.

## Glossary

1. Geocoding Record Linkage Substitution: When the best unique geocoding linkage candidate cannot be identified, either one of the CEL or a less resolute proxy (e.g., postal code area centroid) can be chosen.
2. Residential Geolocation: This is a specific type of record linkage process. In this process, downstream stewards of residential address data (e.g., CCRs) ‘enrich the attribution of a location’ [61] of their data commonly by editing a patient address string reported by the hospital, where the editing entails comparison of a reported address to the ED address point and other georeferenced data. The editing process aspires to an ideal assignment of (x, y) coordinates, whose corresponding address string is discriminant to the ED address point address domain when evaluated with string similarity metrics. Both CCR and ED address point stewards aspire to maximize the discriminant power of their address domains based on string similarity; however, while CCR aspires to assign a unique x,y-coordinate to each unique address, the ED address point stewards are constrained from sharing this goal (e.g., ED address points with different addresses and the same x,y-coordinate can be acceptable to ED address point stewards).
3. Residential Address Discriminant Power: Theoretical construct based on the notion that the domain of residential addresses presents closed-world data (all real-world entities represented in data) and is perfectly discriminant by design.
4. Residential Geolocation Discriminant Power: Discriminant power within the domain of addresses incorporated into ED address points.
5. Candidates Of Equivalent Likelihood: record linkage candidates with the equivalent likelihood of matching to an input string.

## Figures and Tables

**Fig. 3:**
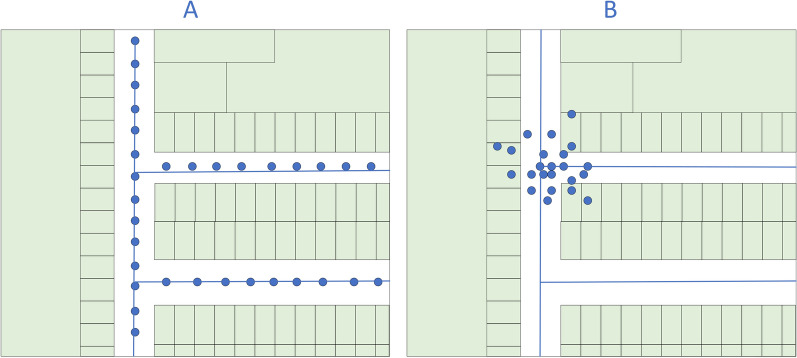
**Street centerline derived ED address point geocodes illustrating proportionate and disproportionate centerline interpolation.** Two hypothetical examples are presented. (A) Centerline geocodes coincide with centerline and their positions are proportionate to house numbers within the address range. This is very common and is included in the category of street-level matches. (B) Centerline geocodes do not coincide with a centerline and their positions are disproportionate to house numbers within the address range. This may indicate a systemic problem with positional accuracy and may invalidate incidence rates at sub-county scales

## Data Availability

Cancer incidence data used in this research are protected information under US HIPAA statute. North Carolina emergency dispatch address points and planimetric data used in this manuscript are generally freely available and can be accessed at NC Center for Geographic Information and Analysis data portal, https://www.nconemap.gov/.

## Appendix

### Appendix 1

#### Hypothetical example of RGDP calculation with different choices of geocoding record linkage substitution

Using a hypothetical example, we illustrate how we determine the number of CEL and calculate RGDP for a patient address. To protect patient confidentiality, we cannot use an example from real data. In this example, the 1101 house number in the patient address record does not exist within Main Street of Town X. At the same time, in ED data, only 3-digit house numbers exist on this street, corresponding to the postal code reported by the patient. Five ED address points correspond to five houses with numbers 110–114, where each house has a single residence. Three ED address points with missing sub-address correspond to one house number 105. Together, the eight ED address points represent the CEL set for the reported patient address, given that all other patient address components are logically consistent with ED address components. Any of the eight CEL can be chosen with equal justification; then the uncertainty in the street-level address is delimited to the eight candidates and therefore, can be captured: RGDP = 1/8 = 0.125. Alternatively, a postal code centroid can be chosen for geocoding, which represents a common practice. As shown later (Table 2), the average count of the CEL within a postal code area is approximately 24,000. Then, RGDP for this geocode is much lower: RGDP = 1/24,000 = 4.1*10^–5^. These two geocoding examples illustrate different geocoding substitution choices and how confidence in residential geolocation quantified as RGDP depends on those choices.

To ensure that RGDP is comparable across cases, we follow the principle of information entropy maximization by distinguishing between discrete and continuous entropy. When uncertainty is delimited because entities in data are designated discrete (e.g., residences) by their statutory stewards, then uncertainty – as viewed by downstream observers – can be captured entirely, because available evidence supports that conclusion. By contrast, there are circumstances when evidence is lacking or contradictory as to whether entities in data are discrete. For example, an apparent orthophoto building is not coincident with the ED address point, or it is not clear that the building is a single residence or even residential. In such a scenario, a non-statutory steward’s downstream assessment of uncertainty can only be estimated. The distinction is important to citizens trying to differentiate authorship (i.e., ED steward or CCR) of uncertainty assessment in cancer incidence data. For residence, quantification of uncertainty by a downstream steward is undertaken as either a neutral observer of discrete entropy (completely captured with CEL) or a partial author of continuous entropy (not completely captured with CEL). Estimation of uncertainty necessitates at least partial authorship (i.e., injection) due to information that the upstream steward may have but the downstream steward may lack. A neutral observer status is more conducive to acquiring citizen trust so that citizens will want to know, for which cases CCRs are neutral observers of uncertainty in residence, or partial authors of it. Given that some cases will inevitably require RGDP estimation, CCRs are incentivized to tabulate and summarize estimated RGDP separately from captured RGDP. For a given case, capture or estimation of RGDP can be recorded in metadata, preferably with a date stamp indicating the time of review. Date stamps are needed because the status of RGDP as captured or estimated can change over time as geographic reference data are updated and revised.

We specifically consider capturing and estimating RGDP in the following conditions. Areas greater than a residential parcel – such as postal code areas – inevitably contain parcels that have [ED address point: residence] cardinality that is [1: (> 1)]. Thus, using postal code centroid as a record linkage substitution choice involves RGDP estimation.

By encoding metadata during geocoding with quantification of confidence in residential geolocation, information entropy can be maximized without compromising flexibility needed in geocode choice, and by extension flexibility in identification of incremental geographic entropy maxima, delimited by either point (CEL) or areas (e.g., building footprint, parcel, or postal code area). An intuitive principle is identifying CEL and the areas of uncertainty or features (building footprint or parcels) that delimit them, and that correspond to the degree of logical consistency of patient address components at record linkage exhaustion. In such a scenario, the analyst has the choice of geocoding to one of the CEL, or to the centroid of the parcel that contains it. Entropy can be maximized with either choice. There are cases for which either choice is preferential; parcel centroids when RGDP is estimated because CEL are missing, and CEL geocodes when RGDP is captured, and parcel centroids do not fall within their associated parcels.

## Appendix 2

### Proposed convention for ed address points derived from vector planimetric features

Within the context of this manuscript, positional accuracy is determined by the association of address point and other vector planimetric features and enumeration areas, which can be verified via geometric coincidence or elimination proximity. In deducing proxy relationships between ED address points and residences they proxy, geometric coincidence is not the only method. Another way to deduce these is with elimination proximity, in which the most proximate ED address point is presumed to be the proxy, with all other ED address points eliminated by comparison. This is especially useful for ED address points derived from street centerline geocodes, relative to parcels containing presumed residence. The capacity of CCRs to assess the positional accuracy of residential geolocation may be limited by the extent of geometric coincidence or elimination proximity of residential geographic reference data. For example, not all ED address points coincide with a building footprint or parcel, or are equally proximate to more than one of these features. In the absence of geometric coincidence or elimination proximity, an interactive review is required to assess positional accuracy. When lack of geometric coincidence rises above a certain threshold, CCRs need to investigate further to screen for systemic steward-specific positional accuracy problems that impact the relationships between the patient's address and other AAs, such as parcels and building footprints. Such systemic problems within ED data may result from the economics of ED data generation. This may require compromises on positional accuracy relative to an intended residence. For example, ED address points are often derived from centroids of vector planimetric features (parcels and digital building footprints), as such a procedure minimizes the number (and expense) of ED address points manually placed. Deriving centroids can also be much cheaper to generate than for example using handheld GPS in the field. Such address points effectively enable the verification (via spatial overlay) of proxy relationships between an ED address point and vector feature and by extension any residence(s) coincident with that feature. The verification of proxy relation between ED address point and parcel or building footprint can break down when ED address points are derived from digital street centerline geocodes, as there may be no geometric coincidence between ED address points and parcel and/or building footprint, or designation of proxied feature is otherwise unclear (Fig. 3). Theoretically, in a subdivision containing single-family detached homes, the number of ED address points associated with this street centerline is equal to the number of parcels, and the correspondence between them is 1:1. Figure 3 illustrates how the relationship between ED address points and parcels can be unclear so that the degree of positional inaccuracy diminishes confidence in the association between ED address points and parcels. Therefore, screening for positional accuracy issues should be conducted as a separate step before calculating RGDP.

If the positional inaccuracy (as depicted by the figure) appears multiple times within the same county, i.e., is systemic to the ED county stewardship, the sub-county incidence rates may be invalid. For example, our ED meta-data (as described later in Step 1, Sect. "Step 1: Identification of CEL for Vector Residential Geographic Data Proxied by ED Address Points") may indicate that in a specific county, 90% of parcels do not coincide with an ED address point while 10% of parcels coincide with th.

e majority of ED address points. Such a scenario indicates a systemic problem in the positional accuracy of ED address points due to data stewardship.

We propose the following convention for geocoding record linkage substitution with the capture of uncertainty as follows:

1. When ED address points are derived from street centerline geocodes with systemic disproportionate interpolation, we presume invalidation of subsequent analysis and recommend interactive review in geocoding record linkage. If the interactive review determines that ED address points’ positional accuracy does not meet geocoding acceptance criteria, we recommend following this with the statutory steward of ED data for further clarification. While awaiting such clarification, we recommend assuming that CEL = 0.
2. When it is determined that street centerlines interpolation is not disproportionate (by pattern recognition of geometric coincidence between ED address points and parcels ED address points) then we rebuttably presume that the cardinality parcel: ED address point reflects real-world conditions, pending record linkage exhaustion. This includes ED address points geocoded to the street centerline with no offset. Then we can use ED address points associated with matching street centerline as CEL to calculate RGDP for a patient address. This circumstance is not presumed to invalidate or reduce confidence in subsequent analyses based on residence (as proxied by ED address point) parcel or enumeration area.
3. When ED address points are derived from parcel centroids, we rebuttably presume that cardinality ED address point: building footprint (via geometric coincidence) reflects real-world conditions. In other words, the intersection of a parcel centroid geocodes with the ED address point geocode is interpreted as a presumptive coincidence of a parcel centroid and a residence. We recommend using ED address points associated with these parcels as CEL for a matching patient address. Such a scenario is not presumed to reduce confidence in subsequent analyses based on residence consisting of building, pending record linkage exhaustion.
4. When ED address points are derived from building footprint centroid in multi-residence parcels, we rebuttably presume that cardinality ED address point: building footprint unit (via geometric coincidence) reflects real-world conditions. In other words, the intersection of a building footprint centroid geocodes with the ED address point geocode is interpreted as a presumptive coincidence of a building footprint centroid and a residence. We recommend using ED address points associated with the multi-residence parcel as CEL. Such a scenario is not presumed to reduce confidence in subsequent analyses based on residences consisting of building units, pending record linkage exhaustion.
